# Massive hemoptysis and deep venous thrombosis presenting in a woman with Hughes-Stovin syndrome: a case report

**DOI:** 10.1186/1752-1947-4-109

**Published:** 2010-04-21

**Authors:** Hamdan Al-Jahdali

**Affiliations:** 1 Medical Department, King Saud University for Health Sciences, King Abdulaziz Medical City, Riyadh, 11426, Saudi Arabia

## Abstract

**Introduction:**

Hughes-Stovin syndrome is a very rare disease with fewer than 30 cases reported in the literature. The disease is thought to be a variant of Behcet's disease and is defined by the presence of pulmonary artery aneurysm in association with peripheral venous thrombosis.

**Case presentation:**

A previously healthy 23-year-old Saudi woman presented with massive hemoptysis a day prior to her admission to our hospital. She had a six-month history of recurrent fever, cough, dyspnea, and recurrent oral ulceration. Contrast-enhanced computed tomography scan of her chest and pulmonary angiogram demonstrated a single right-lower lobe pulmonary artery aneurysm. She underwent thoracotomy and right lower lobe resection. Her postoperative course was complicated by deep vein thrombosis. She also developed headache and papilledema, while a magnetic resonance imaging of her brain suggested vasculitis. Based on these clinical presentations, she was diagnosed and treated with Hughes-Stovin syndrome.

**Conclusion:**

The majority of cases of Hughes-Stovin syndrome are reported among men, with only two cases occurring in women. A case of Hughes-Stovin syndrome occurring in a woman is presented in this report. She was treated successfully with multimodality treatment that includes surgery, steroids and cytotoxic agents.

## Introduction

The combination of pulmonary artery aneurysm and thromboembolic disease is uncommon but is reported in association with Behcet's disease [1-5]. The disease affects mainly adults, especially men [1-3]. It is prevalent in Japan, the Middle East, and the Mediterranean but it is also found worldwide [1-4]. Behcet's disease is a form of systemic vasculitis affecting mainly the venules [1-4]. No laboratory tests are diagnostic of Behcet's disease; hence the diagnosis is made based on clinical criteria. The patient must have recurrent oral ulceration with at least two of the following: recurrent genital ulceration, eye lesions, skin lesions, or a positive pathergy test [1,6].

In 1911, Beattie and Hall reported the association between multiple aneurysms of the pulmonary arteries and venous thrombosis of the lower limbs [7]. The same combination was reported later by Hughes and Stovin in 1959 [8]. They reported four cases of deep venous thrombosis and multiple segmental pulmonary artery aneurysms. Since then, this association has been named Hughes-Stovin syndrome. Hughes-Stovin syndrome occurs very rarely, with fewer than 30 cases reported in the literature [9,10]. It affects mainly men, with only two cases describing women [11]. Patients usually present with fever, chills, dyspnea, cough, hemoptysis, and venous thrombosis [9,10].

The main cause of death in Hughes-Stovin syndrome is massive hemoptysis secondary to the rupture of A pulmonary artery aneurysm [9,10,12]. The pathogenesis of Hughes-Stovin syndrome is unclear, although many hypotheses have been made to explain the manifestations of this syndrome. It has been suggested that pulmonary artery aneurysms may arise from a degenerative defect in the bronchial arteries or may be mycotic in origin resulting from emboli infected with low-grade virulence organisms. It may also be due to angiodysplasia of the bronchial arteries [9,11]. However, none of these hypotheses are widely accepted.

It is currently thought that Hughes-Stovin syndrome is a form of vasculitis similar to Behcet's disease [5,9,13,14]. In reality, Behcet's disease and Hughes-Stovin syndrome are the only vasculitides known to cause pulmonary artery aneurysms in patients [1,3,12]. Many authors have even suggested that Hughes-Stovin syndrome may represent a variant of Behcet's disease [3,5,15].

## Case presentation

**Figure 1:**
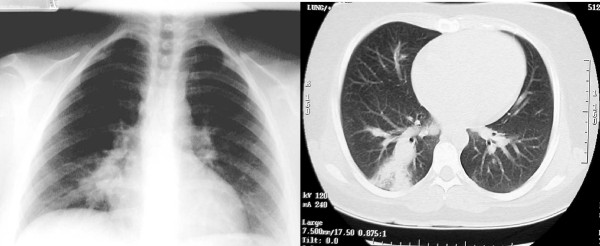
**Chest radiograph revealed an ill-defined rounded infiltrate in the right lower lobe (white arrow)**.

**Figure 2:**
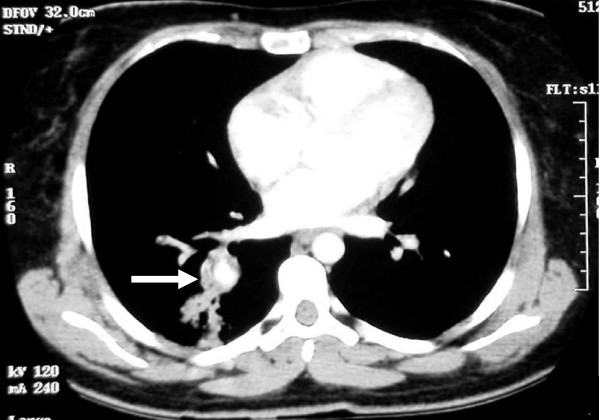
**Contrast-enhanced computed tomography scans of the chest demonstrated a right lower lobe pulmonary artery aneurysm (white arrow)**.

A previously healthy 23-year-old Saudi woman presented with massive hemoptysis a day before she was admitted to our hospital. She had a six-month history of recurrent fever, cough, dyspnea, and recurrent oral ulceration. Her physical examination was within normal limits. An initial blood work-up showed that she had mild leukocytosis (14.7 × 10^9^/L) and elevated erythrocyte sedimentation rate (85 mm/hr). Chest X-ray and computed tomography (CT) scan revealed an ill-defined rounded infiltrate in her right lower lobe (Figures 1A and 1B). Contrast-enhanced CT scans of her chest demonstrated a right lower lobe pulmonary artery aneurysm (Figure 2).

Because she complained of intermittent headache over the past six months, a CT scan of the brain with contrast was done. It revealed no abnormalities. However, a magnetic resonance imaging (MRI) scan of the brain showed increased high-intensity signals bilaterally especially in the gray/white matter junction (>4 foci) in fluid-attenuated inversion recovery (FLAIR) images. This was highly suggestive of vasculitis.

**Figure 3:**
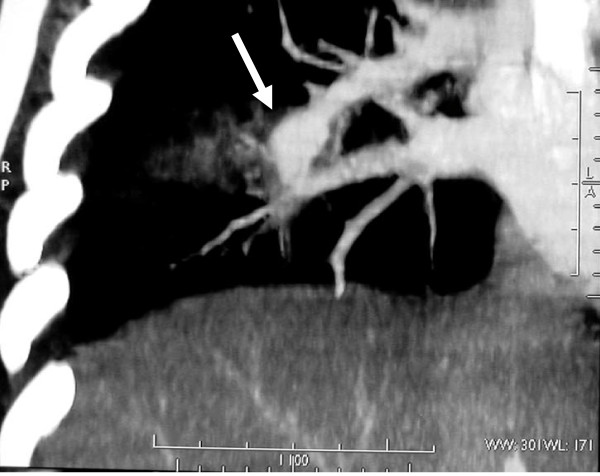
**Cardiac magnetic resonance imaging showed the aneurysm in the right interlobar pulmonary artery (white arrow)**.

Transthoracic echocardiography showed a 1.8 cmx1.6 cm non-mobile right ventricular mass attached to her interventricular septum. Transesophogeal echocardiography showed the same mass having a texture similar to her papillary muscle. A cardiac MRI was subsequently done, which showed a right ventricular mass with the same signal of the cardiac muscle. An aneurysm in her right interlobar pulmonary artery was also seen (Figure 3). Further laboratory workups ruled out other connective tissue diseases except for elevated lupus anticoagulant level (LA1 = 54.9, normal = 30 to 44; LA2 = 37.2, normal = 26 to 32).

**Figure 4:**
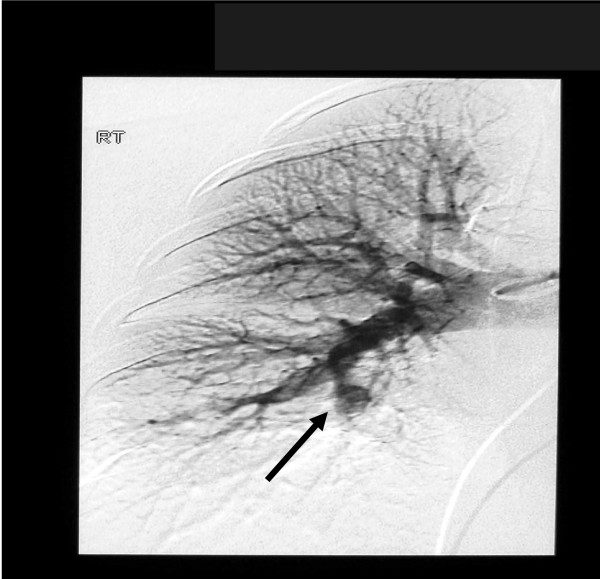
**Pulmonary angiography showed a single aneurysm of the right interlobar pulmonary artery (black arrow)**.

Our patient underwent pulmonary angiography to rule out other aneurysms. The procedure showed a single aneurysm of her right interlobar pulmonary artery. (Figure 4).

She then underwent right thoracotomy with the removal of her right lower lobe. Pathological examination revealed multifocal arterial thrombosis with marked luminal narrowing, partial destruction of her arterial wall, and marked intimal fibrosis (fibroelastosis).

Our patient's postoperative course was uneventful. A few days later, however, she developed swelling in her right lower limb. Doppler sonography revealed deep vein thrombosis in her right iliac, right common femoral, right superficial femoral and right popliteal veins. Spiral CT scan of her chest showed a small filling defect in her right apical segmental artery consistent with pulmonary embolism. She was thus commenced on intravenous unfractionated heparin followed by oral coumadin.

An ophthalmologic examination of our patient showed no evidence of iritis or retinal vasculitis. However, she was found to have optic disc swelling (papilledema). She was thus diagnosed with Hughes-Stovin syndrome, which is a variant of Behcet's disease. She was treated with methylprednisolone 1 gm intravenously for five days. She then had 50 mg/day of oral azathioprine. Her dosage increased gradually to 150 mg/day, with 0.5 mg bid of colchicines. A repeat MRI of her brain three weeks after the treatment showed complete resolution of the high intensity signals in FLAIR images, thus indicating her favorable response to treatment. Repeat spiral CT scans of her chest for the succeeding 12 months revealed no recurrence of pulmonary aneurysm.

## Discussion

Hughes-Stovin syndrome is considered a variant of Behcet's disease [3,5]. Both diseases are characterized by the destruction of the wall of the pulmonary arteries and perivascular infiltration. Nearly 25% of patients with Hughes-Stovin syndrome develop vascular thromboembolism, arterial aneurysms, and arterial and venous occlusions with nonspecific vasculitis. The vascular lesions are arterial in 7%, venous in 25%, and both in 68% of reported cases [1,4,16]. Arterial aneurysm is often associated with the poor prognosis of patients, and is usually found present in the pulmonary arteries and in the aorta [1,4,12,16].

Pulmonary involvement is seen in 1% to 7% of reported cases. Pulmonary lesions, which are seen in Behcet's disease, are pulmonary arterial aneurysms, arterial-venous thrombosis, pulmonary infarcts, focal atelectasis, and occasionally pleural effusions. Pulmonary vasculitis is multifocal and thrombosis is seen in the branches of pulmonary arteries [1,2,4].

Our patient described in this case report has only a single aneurysm. Aneurysms may be single or multiple, unilateral or bilateral. It is rarely multiple or bilateral. Reports indicate that pulmonary lesions and deep venous thrombosis of the lower extremities are the most frequent findings [5,17]. The exact mechanism of thrombosis in Behcet's disease is unknown. Thrombophilia does not seem to play a major role in the notable tendency among patients with Behçet's disease to develop thrombosis. However, hyperhomocysteinemia is also assumed to be an independent factor in the development of venous thrombosis [18,19].

## Conclusion

In the patient described in this case report, anticoagulants were used to safely treat her deep vein thrombosis after her pulmonary artery aneurysm was resected (right lower lobectomy). Surgical resection has also been performed in some cases. However, it may be difficult to perform it if there are multiple or bilateral aneurysms [9]. The embolization of pulmonary artery aneurysms has been reported by many authors [5,9,11,17]. The advantages of embolization are that it is less invasive and has the ability to treat multiple and bilateral aneurysms. Corticosteroids, alone or in combination with cytotoxic drugs, have been tried in patients with Hughes-Stovin syndrome [4,9,17]. Although found to be effective in some cases, they were not always useful in preventing disease progression particularly in the late stages of the disease [20].

Our patient described in this case report underwent right lower lobectomy with an uneventful postoperative course. She was treated with pulse steroid in combination with azathioprine. She responded well to treatment and remains in complete remission.

## Consent

Written informed consent was obtained from our patient for publication of this case report and any accompanying images. A copy of the written consent is available for review by the Editor-in-Chief of this journal.

## Competing interests

The author declares that they have no competing interests.
